# Ancient Ecuadorian genomes reveal millennia of genetic continuity on the Pacific Coast of South America

**DOI:** 10.1093/pnasnexus/pgag323

**Published:** 2026-10-27

**Authors:** John Lindo, Daniel Rivas Alava, Sara Juengst, Yemko Pryor, Sophie K Joseph, Juan J Ortiz Aguilu, Richard Lunniss

**Affiliations:** Department of Anthropology, University of California, Berkeley, CA 94720, USA; Department of Anthropology, Emory University, Atlanta, GA 30322, USA; Department of Anthropology, University of North Carolina at Charlotte, Charlotte, NC 28223, USA; Department of Anthropology, University of Michigan, Ann Arbor, MI 48109, USA; Department of Molecular and Cell Biology, University of California, Berkeley, Berkeley, CA 94720, USA; Laboratorio y Centro de Análisis e Investigación Arqueológica, Üniversidad Interamericana De Puerto Rico - Recinto De Barranquitas, Barranquitas 00794, Puerto Rico; Instituto de Investigación, Universidad Tecnica de Manabí, Portoviejo 130105, Ecuador

## Abstract

The demographic history of pre-Columbian South American populations remains poorly understood, particularly along the Pacific coast where ancient genomic data are scarce. Here, we present the first whole genomes from ancient individuals buried at Salango, Ecuador, spanning ∼2,300 to ∼740 years before present and representing 3 distinct cultural phases. Despite major environmental disruption following the Pululahua volcanic eruption and substantial sociopolitical change, these individuals exhibit remarkable genetic continuity across nearly 2 millennia. Population genetic analyses consistently support long-term continuity at Salango, while demographic modeling suggests that the Ecuadorian coastal lineage diverged from other Southern Native American (SNA) lineages approximately 9,800 years ago (95% CI: 9.6–10.1 ka). Together, these findings are consistent with coastal Ecuador representing a long-standing component of early South American population structure while maintaining connections with neighboring Andean populations through time. In addition, we recovered authentic *Mycobacterium tuberculosis* complex (MTBC) DNA from an infant dated to ∼2,300 BP with skeletal lesions consistent with tuberculosis, providing molecular evidence for one of the earliest documented cases of MTBC infection in pre-contact South America. Although the moderate pathogen genome coverage precludes detailed phylogenetic interpretation, the combined osteological and molecular evidence expands the record of tuberculosis in ancient South America. Overall, our study provides the first ancient genomic data from Ecuador, establishing an important geographic reference for investigating the demographic and biological history of the Pacific coast of South America.

Significance Statement Ancient genomes from Salango provide the first genomic evidence from Ecuador's Pacific coast, revealing over 1,500 years of genetic continuity despite major environmental and cultural transformations. These findings indicate coastal Ecuador as an important component of early Southern Native American population history and highlight long-term connections between coastal and Andean populations. We also recovered authentic *Mycobacterium tuberculosis* complex DNA from an infant with skeletal lesions consistent with tuberculosis, providing rare molecular evidence of infectious disease in pre-contact Ecuador. Together, these results offer new insights into the demographic and biological history of the Pacific coast of South America.

## Introduction

The human settlement of South America unfolded rapidly after the initial peopling of the continent, yet the geographic distribution of ancient genomic data remains uneven. Ancient genomic research has expanded considerably in parts of the Central Andes, including the Peruvian highlands and from the Peruvian and northern Chilean coasts. Ecuador, however, remains a major gap, with no previously published ancient genomes from either the Ecuadorian coast or the Ecuadorian highlands. Although present-day Indigenous genomes from the Ecuadorian highlands provide important comparative context, they cannot substitute for direct temporal comparisons with ancient highland populations. Situated at the intersection of the northern Andean highlands and the Pacific coast, Ecuador offers an important setting for investigating population continuity and interaction between these ecologically and culturally distinct but historically connected regions. In particular, the Ecuadorian coast—an early center of agriculture, trade, and ritual integration—provides a critical yet genomically underrepresented context for understanding the population history of Ecuador and the broader Central Andes.

Archaeological evidence reveals a deep sequence of human occupation along Ecuador's littoral zone beginning with the terminal Pleistocene *Las Vegas* culture (ca. 9,000–5,300 BCE) (1, 2). The subsequent Formative period traditions of Valdivia, Machalilla, and Chorrera, together with their regional variants, pioneered pottery, permanent village life, and ceremonial architecture while materializing complex cosmological systems (3, 4). By the Regional Development period, the central coast was home to politically and ritually diverse chiefdom societies—including Guangala, Bahía, and Jama-Coaque—that exhibited increasing social complexity and regional interaction (5, 6). Emerging from Guangala and Bahía traditions, the Integration period Manteño culture (ca. 650–1,530 CE) (7) was characterized by demographic expansion, the consolidation of chiefly authority, intensive agriculture, the construction of stone architecture (including intertidal fish traps), and a far-reaching maritime exchange network centered on *Spondylus* shell trade (8).

The site of Salango (Figure 1A), located on Ecuador's central coast, encapsulates much of this long cultural continuum. Human occupation began with a Late Archaic fishing camp around 4000 BCE (9), marking the start of over 6,000 years of largely continuous settlement sustained by fishing and shellfish collection. The earliest burials are associated with the Machalilla fishing village (1,500–900 BCE) (10, 11). During the Engoroy phase—the local variant of Chorrera (900–330 BCE)—the settlement transitioned into a ritual sanctuary increasingly centered on the veneration of ancestors and the human dead (9, 12).

**Figure 1:**
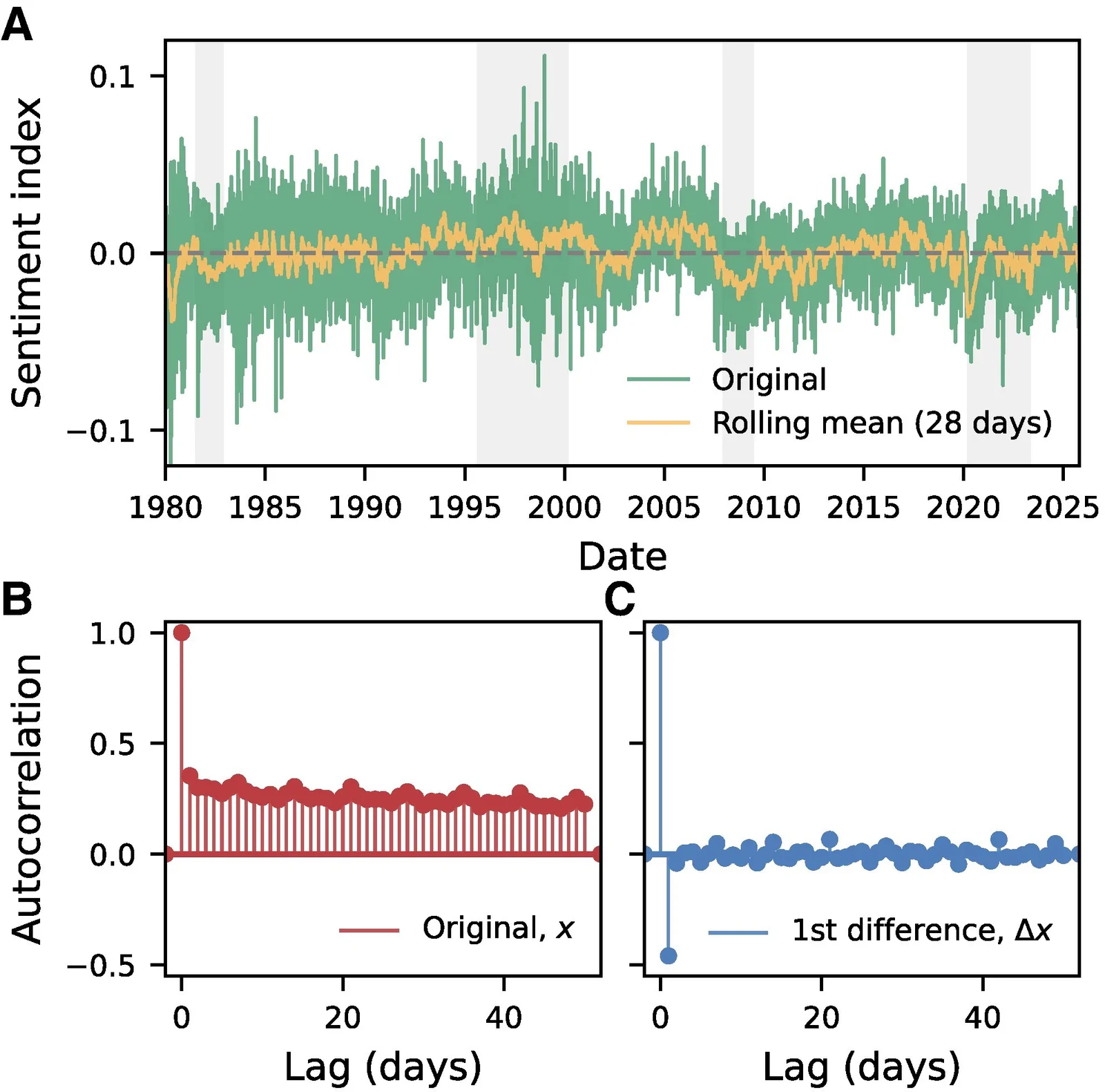
Geographic context and genetic affinities of ancient individuals from Salango, Ecuador. (A) Map of Ecuador showing the archaeological site of Salango (blue star) located on the central Pacific coast. (B) Principal component analysis (PCA) of genome-wide SNP data from ancient and present-day Indigenous American individuals. Low-coverage ancient genomes were projected onto principal components defined by present-day Indigenous populations. Ancient Salango individuals (magenta) form a tight cluster, with their nearest neighbors along PC1 corresponding to populations from Ecuador and Colombia. The inset highlights the cluster of Central and South American samples.

The catastrophic Pululahua volcanic eruption (∼330 BCE) abruptly ended this Formative tradition. A brief Very Early Guangala phase (∼300–200 BCE) followed, marked by an unusual burial program dominated by infants and children and featuring radically novel funerary treatments (13, 14). Subsequently, the site was transformed into an elaborate Early Regional Development (ca. 200 BCE–300 CE) elite burial complex that served as a nexus between Bahía II and Early Guangala cultural traditions (14). The central funerary enclosures incorporated large stone anchors and other evidence suggesting the innovation of large ocean-going balsa rafts, while additional artifacts point to sustained exchange with both the Ecuadorian highlands and the north Peruvian coast.

During the Middle Guangala phase (300–600 CE), Salango was repurposed: the sanctuary lost its funerary role but acquired a perimeter wall accompanied by massive offerings of fish and select artifacts (15). The following centuries are less clearly documented, yet by the Late Manteño phase (ca. 1,200–1,500 CE) (16), Salango had developed into a key node within the *Señorío de Salangome* (17), with its mainland settlement functioning as a port engaged in long-distance maritime exchange and its island counterpart serving ritual and ceremonial purposes (18).

Yet, despite this rich cultural record, genomic data from ancient Ecuadorian populations have been entirely lacking—leaving unresolved questions about demographic continuity, post-volcanic population recovery, and coastal–highland interaction through time. Here, we present the first genome-wide data from ancient individuals excavated at Salango, dating to the Very Early Guangala (∼2,300 BP), Early Guangala (∼1,900 BP), and Early Manteño (∼740 BP) phases. These individuals provide a unique window into population continuity and transformation across 2 millennia of Ecuadorian prehistory, linking archaeological evidence of cultural persistence with genetic signatures of demographic stability and interaction.

In addition to providing insights into population history, the Salango remains offer a rare opportunity to investigate infectious disease in ancient coastal Ecuador. One infant burial from the Very Early Guangala phase (∼300–200 BCE) exhibited skeletal lesions consistent with tuberculosis (TB) (13). Differential diagnosis and lesion patterning are most consistent with congenital TB or tuberculous meningitis, representing the earliest documented case of TB in Ecuador and among the few reported infant cases in the pre-contact Americas. The recovery of ancient DNA from this individual provides an opportunity to complement the osteological evidence with molecular analyses, offering independent support for the presence of *Mycobacterium tuberculosis* complex (MTBC) infection in ancient coastal Ecuador.

## Results

### Ancient DNA recovery and authentication

We successfully sequenced 4 ancient genomes from 4 individuals excavated at the site of Salango on Ecuador's central coast, spanning 3 cultural horizons: the Very Early Guangala (ca. 2,300 BP), Early Guangala (ca. 1,900 BP), and Early Late Manteño (ca. 740 BP) phases. The genomes ranged from 0.54× to 2.31× mean coverage (Table 1). All samples exhibited nucleotide misincorporation patterns characteristic of postmortem DNA damage, including elevated cytosine-to-thymine substitutions at 5′ termini (Figure S1). Estimates of mitochondrial contamination were uniformly low (<4%; Table 1), and X-chromosome-based contamination in males was negligible. Mitochondrial haplotypes for all individuals fall within lineages common among Indigenous populations of the Americas, including subclades of haplogroups A2, B2, and D1, consistent with established South American maternal diversity. No evidence of non-Indigenous haplogroups was detected. Together, these data confirm the integrity of the Salango genomes and provide a robust foundation for exploring genetic continuity and population structure through time along Ecuador's Pacific coast.

**Table 1:** Sample information.

ID	mtDNA haplogroup	Contamination estimate	Mean coverage	Dating	Archaeological site	Cultural phase
SLG-9B	D1	0.025	2.318181818	2,300 BP^a^	Salango, Ecuador	Very Early Guangala
SLG-12B	A2	0	1.927272727	2,300 BP^a^	Salango, Ecuador	Very Early Guangala
SLG-27B	A2	0.031	0.888636364	740 BP^b^	Salango, Ecuador	Early Manteño
SLG-33B	B2	0.035	0.543636364	1,900 BP^a^	Salango, Ecuador	Early Guangala

Contamination estimates and mitochondrial haplogroups were calculated with Haplogrep 3 (19).

^a^Culturally dated.

^b^Directly dated; *see* Table S2.

### Population structure and genetic affinities

To investigate genetic affinities and assess potential population continuity across cultural phases, we performed a principal component analysis (PCA) using genome-wide SNP data from ancient and present-day Indigenous American populations, with Siberian samples included as outgroups (Table S1). Because the Salango genomes were of low coverage, they were projected onto principal components defined by higher-coverage reference individuals. In the resulting plot (Figure 1B), all Salango individuals (SLG9B, SLG12B, SLG27B, SLG33B) cluster closely together, indicating genetic homogeneity within the site despite temporal separation. The cluster occupies a position near ancient and present-day populations from Ecuador, Colombia, and Peru, consistent with shared ancestry within a northwestern South American genetic continuum. Along PC1 (18.2%) and PC2 (10.9%), the Salango cluster is clearly separated from North American and Siberian groups but shows proximity to both coastal and highland Andean populations, suggesting enduring regional interaction and continuity through time.

An additional PCA restricted to Indigenous people from the Americas provided greater resolution of regional structure and incorporated 4 ancient individuals from the Peruvian coast (Figure S2; *see* Methods). The Peruvian coastal individuals fell within the broader distribution of ancient Central Andean populations, while SLG9B, SLG12B, and SLG27B remained broadly associated with the northwestern South American variation represented by ancient Ecuadorian, Colombian, and Andean individuals across PC1–PC4. Although SLG33B appears more separated in the regional PCA, only 664 SNPs were available for its projection. Consequently, its position is likely unstable and was not interpreted as evidence of demographic differentiation. Overall, the regional analysis supports the affinities observed in the continental PCA and indicates that the placement of the Salango individuals is not solely a consequence of the inclusion of North American and Siberian samples.

To formally assess the shared ancestry patterns suggested by the PCA, we computed outgroup *f_3_*-statistics of the form *f_3_(Mbuti; X, SLG)*, where *X* represents ancient or present-day Indigenous populations from the Americas (Figure 2). This statistic quantifies the amount of shared genetic drift between each Salango genome and comparison populations since their divergence from an African outgroup, providing a robust measure of genetic affinity. Because it is relatively insensitive to missing data, the *f_3_*-statistic is particularly well suited for low-coverage ancient genomes.

**Figure 2:**
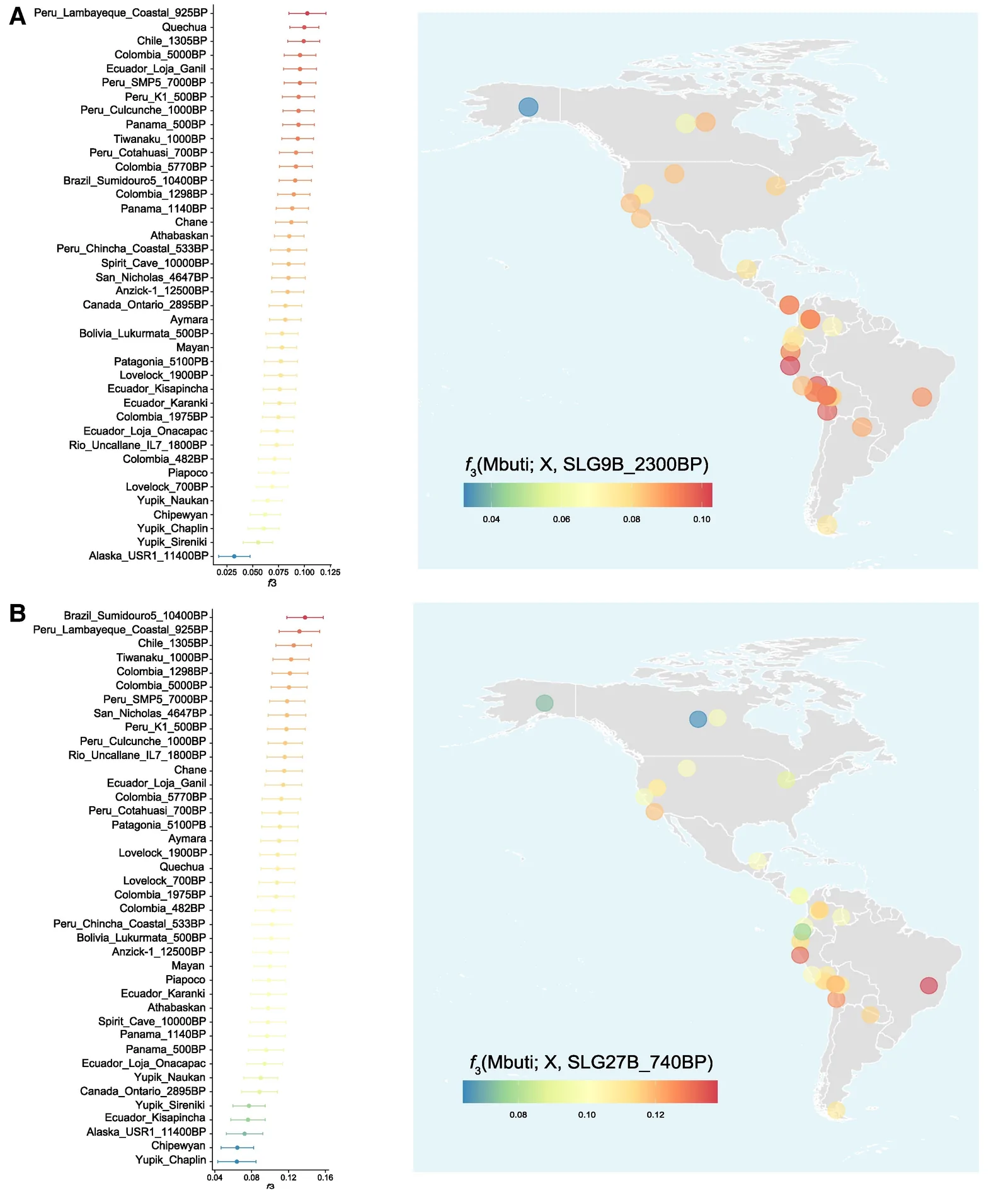
Shared genetic drift of ancient individuals from Salango with ancient and present-day Indigenous American populations. Statistics of the form *f*_3_(Mbuti; X, SLG) were calculated for (A) the Very Early Guangala individual SLG-9B (∼2,300 BP) and (B) the Early Manteño individual SLG-27B (∼740 BP). Points and horizontal bars show f_3_ estimates and their associated uncertainty, respectively. The accompanying maps show the geographic distribution of the comparison populations, with point colors corresponding to *f*_3_ values according to the scale shown in each panel. Warmer colors indicate greater shared genetic drift. In panel (A), SLG-9B shows its highest affinities with Peru_Lambayeque_Coastal_925BP, Quechua, Chile_1305BP, Colombia_5000BP, Ecuador_Loja_Ganil, and Peru_SMP5_7000BP, indicating shared genetic drift with populations spanning the Pacific coast, Andean highlands, and adjacent regions. In panel (B), SLG-27B shows its highest affinities with Brazil_Sumidouro5_10400BP, Peru_Lambayeque_Coastal_925BP, Chile_1305BP, Tiwanaku_1000BP, Colombia_1298BP, Colombia_5000BP, Peru_SMP5_7000BP, and San_Nicholas_4647BP. Together, the results place both Salango individuals within a broad western South American genetic continuum, while the differing rankings suggest temporal changes in the relative strength of affinities among coastal, Andean, and more geographically distant populations.

For the Very Early Guangala individual SLG9B (2,300 BP), the highest outgroup-*f*_3_ point estimate was obtained with the Lambayeque-associated individual from El Brujo on the northern Peruvian coast (925 BP) (20), followed by present-day Quechua and ancient individuals from Chile, Colombia, Ecuador, and the Peruvian highlands. Other relatively high estimates included Colombia_5000BP (21), Ecuador_Loja_Ganil (22), Peru_SMP5_7000BP, Peru_K1_500BP (23), and Peru_Culcunche_1000BP (24). The analysis indicates broad shared ancestry with populations distributed across the northern and central Andes and the Pacific coast, rather than an affinity restricted primarily to early Holocene populations. Because the standard errors overlap substantially among many comparisons, we interpret the ordering of the estimates descriptively and do not consider the highest-ranked populations to be statistically distinguishable. A similar pattern is observed for SLG-12B (∼2,300 BP), recovered from the same burial complex at Salango (Figure S5A). The highest point estimate was obtained with the Chincha coastal individual from Peru (∼533 BP) (25), followed by a contemporary individual from Loja, Ecuador, Colombia_1298BP, Brazil_Sumidouro5_10400BP (26), San_Nicholas_4647BP (27), and Anzick-1_12500BP (28). Additional relatively high estimates involved populations from Peru, Chile, Colombia, and the wider Americas. Although Peru_Chincha_Coastal_533BP was buried in the middle Chincha Valley on the southern Peruvian coast, genomic analysis indicates that this individual belonged to a group with substantial northern Peruvian coastal ancestry (25). The prominent affinity between SLG12B and Peru_Chincha_Coastal_533BP therefore provides evidence of genetic connections between Salango and populations associated with the northern Peruvian coastal ancestry.

The Early Manteño individual, SLG27B (∼740 BP), also exhibits a geographically broad affinity profile. The highest point estimate was obtained with Brazil_Sumidouro5_10400BP, followed by the Lambayeque-associated individual from El Brujo in coastal Peru. Other relatively high estimates involved ancient individuals from Chile, Tiwanaku, Colombia, and Peru, including Peru_SMP5_7000BP, San_Nicholas_4647BP, Peru_K1_500BP, and Peru_Culcunche_1000BP. The prominence of the El Brujo comparison for both SLG9B and SLG27B, together with the high Chincha estimate for SLG12B, indicates that northern Peruvian coastal-related ancestry is represented among the stronger affinities of all 3 Salango individuals.

Taken together, the outgroup-*f*_3_ results reveal broadly overlapping affinity profiles among individuals from the Very Early Guangala and Early Manteño phases. All 3 share genetic drift with populations distributed across the Andes and Pacific coast, while the El Brujo and Chincha comparisons provide more direct evidence of connections with Peruvian coastal populations. These findings are consistent with long-term genetic relationships linking coastal Ecuador, northern Peru, and adjacent Andean regions, rather than a discrete transition from early pan-continental ancestry during the Guangala period to exclusively regional ancestry during the Manteño period.

To further explore the ancestry relationships indicated by the *f_3_* results, we computed *f_4_*-statistics of the form *f_4_(*Mbuti, SLG; Athabaskan, X*)*, where X represents ancient or present-day Indigenous populations from the Americas (Figure 3). The *f_4_* statistic tests for deviations from a strictly tree-like relationship by measuring the covariance of allele frequency differences between 2 pairs of populations. Athabaskan was used as a post-NNA (Northern Native American) reference to anchor the comparison and to assess asymmetry in allele sharing between Salango and other lineages after the NNA–SNA divergence.

**Figure 3:**
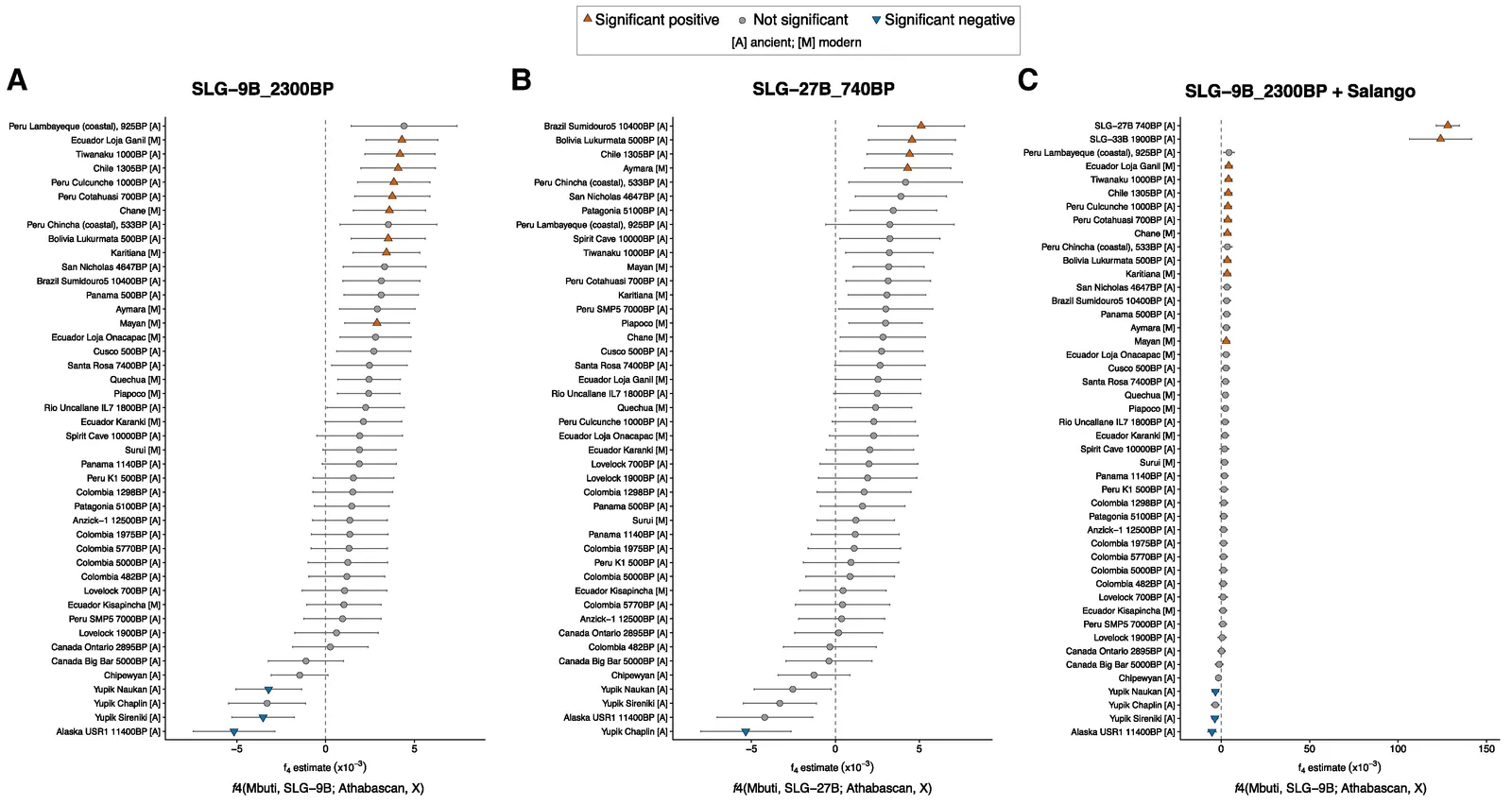
Symmetry tests of allele sharing among ancient individuals from Salango and Indigenous populations of the Americas. Statistics have the form *f*4(Mbuti, Salango individual; Athabaskan, X), where X is the comparison population. (A) SLG-9B (∼2,300 BP). (B) SLG-27B (∼740 BP). (C) SLG-9B with SLG-27B and SLG-33B included as additional Salango comparison individuals. Points indicate f4 estimates and horizontal bars indicate 95% confidence intervals. Positive values indicate greater allele sharing between the Salango individual and X relative to Athabaskan, whereas negative values indicate comparatively greater allele sharing between the Salango individual and Athabaskan. Upward triangles indicate significant positive values (Z > 3), circles indicate nonsignificant values, and downward triangles indicate significant negative values (Z < −3). Labels identify ancient ([A]) and modern ([M]) comparison populations.

For the Very Early Guangala individual SLG-9B (∼2,300 BP) (Figure 3A), the *f*_4_ analysis shows significant positive values with Tiwanaku_1000BP, Ecuador_Loja_Ganil, Chile_1305BP, Peru_Culcunche_1000BP, Karitiana, Peru_Cotahuasi_700BP, Chane, Bolivia_Lukurmata_500BP, and Mayan. This pattern indicates that SLG-9B shares more alleles with a geographically extensive set of Andean, coastal, and present-day Indigenous populations than with Athabascan. Demographically, these affinities are consistent with SLG-9B belonging to a broader Southern Native American ancestry network that extended across the Pacific coast, the central Andes, and adjacent lowland regions. They may reflect shared ancestry and long-term regional population structure, as well as connectivity across these regions.

SLG-12B (∼2,300 BP), recovered from the same burial phase, shows a related but more limited pattern (Figure S5B). Significant positive values occur with Chile_1305BP, Karitiana, Mayan, and Bolivia_Lukurmata_500BP, whereas significant negative values occur with Yupik_Naukan, Yupik_Sireniki, Yupik_Chaplin, and Alaska_USR1_11400BP. The broadly concordant signals for SLG-9B and SLG-12B suggest that individuals from the same early burial context shared a similar underlying ancestry profile and were embedded in comparable regional genetic networks. The positive affinities with Bolivia_Lukurmata and Chile are consistent with connections extending between the Ecuadorian coast and the Andean highlands or southern Pacific coast, while the affinities with Karitiana and Mayan indicate that the observed pattern is not restricted to a single local or highland population. These results are compatible with population continuity at Salango during the Very Early Guangala phase, alongside wider interregional connections.

The Early Manteño individual SLG-27B (∼740 BP) exhibits significant positive *f*_4_ values with Brazil_Sumidouro5_10400BP, Bolivia_Lukurmata_500BP, Chile_1305BP, and Aymara (Figure 3B). These results indicate greater allele sharing between SLG-27B and both early South American and later Andean populations relative to Athabascan. The affinities with Bolivia_Lukurmata and Aymara are consistent with connections involving the Andean highlands, while the Chilean comparison supports broader links along the western South American corridor. The significant affinity with Brazil_Sumidouro5_10400BP is also consistent with the strong outgroup-*f*_3_ affinity observed for SLG-27B and suggests that some ancestry shared with early Southern Native American lineages persisted into the late pre-contact period. In demographic terms, the SLG-27B results are consistent with continued local ancestry at Salango accompanied by increased integration into wider coastal and highland interaction networks during the Manteño period.

To evaluate continuity directly across the Salango temporal sequence, we calculated *f*_4_(Mbuti, SLG-9B; Athabascan, X), with SLG-27B and SLG-33B as comparison populations (Figure 3C). Both comparisons produce very large positive values, indicating substantially greater allele sharing between SLG-9B and the other Salango individuals than between SLG-9B and Athabascan. This result provides direct evidence that the Salango individuals retained substantial shared ancestry across approximately 1,500 years, spanning the Very Early Guangala and Early Manteño phases. The persistence of this shared ancestry despite major cultural and environmental changes suggests demographic continuity at the site, while the broader *f*_3_ and *f*_4_ patterns indicate that continuity occurred within an increasingly interconnected western South American population landscape. However, the SLG-33B comparison should be interpreted cautiously because of its low overlapping SNP count (File S1).

Additional *f*_4_ analyses using South and Central American reference populations were also performed (Figures S6–S10; File S1). Non-Athabascan temporal contrasts and highland–coastal comparisons did not show significant asymmetry at |Z| > 3, indicating that these tests do not resolve strong allele-sharing differences among the tested regional populations. In contrast, Sumidouro5-specificity analyses consistently favored greater relative affinity with Brazil_Sumidouro5 than with the alternative reference populations. Taken together, the *f*_4_ results support substantial continuity among the Salango individuals while also revealing changing, and partly population-specific, connections with Andean, coastal, lowland, and broader Southern Native American populations.

### Demographic model

We next fit a demographic model with fastsimcoal2 (29) using a joint SFS that included SLG, Rio Uncallane (23) (Titicaca Basin), ancient Colombia, Patagonia, and Brazil (Sumidouro), with USR1 (30) (Alaska) and MA-1 (31) (Siberia) as outgroups. To anchor deep events, we fixed the Siberia→Americas split (30) at ∼23 ka and the NNA/SNA divergence at ∼16 ka, consistent with prior genomic syntheses that place the NNA–SNA split ∼17.5–14.6 ka (26, 27) and emphasize a rapid southward spread of SNA ancestry into South America soon thereafter.

Under the archaeologically constrained parameterization, the best-fitting topology recovered a sequential series of South American divergences (Figure 4). Across 100 parametric bootstrap re-estimations, the median inferred divergence between the SLG and Rio Uncallane lineages was 9.30 ka (95% bootstrap interval: 9.18–9.38 ka), followed by the divergence of SLG from the broader SNA lineage at 9.80 ka (9.63–10.10 ka). The corresponding median estimates for the Patagonia, Brazil/Sumidouro, and Colombia divergences were 11.33 ka (11.20–11.33 ka), 11.83 ka (11.73–11.88 ka), and 12.30 ka (12.18–12.30 ka), respectively. These empirical intervals describe uncertainty conditional on the specified topology, fixed deep divergence times, and archaeological calibration.

**Figure 4:**
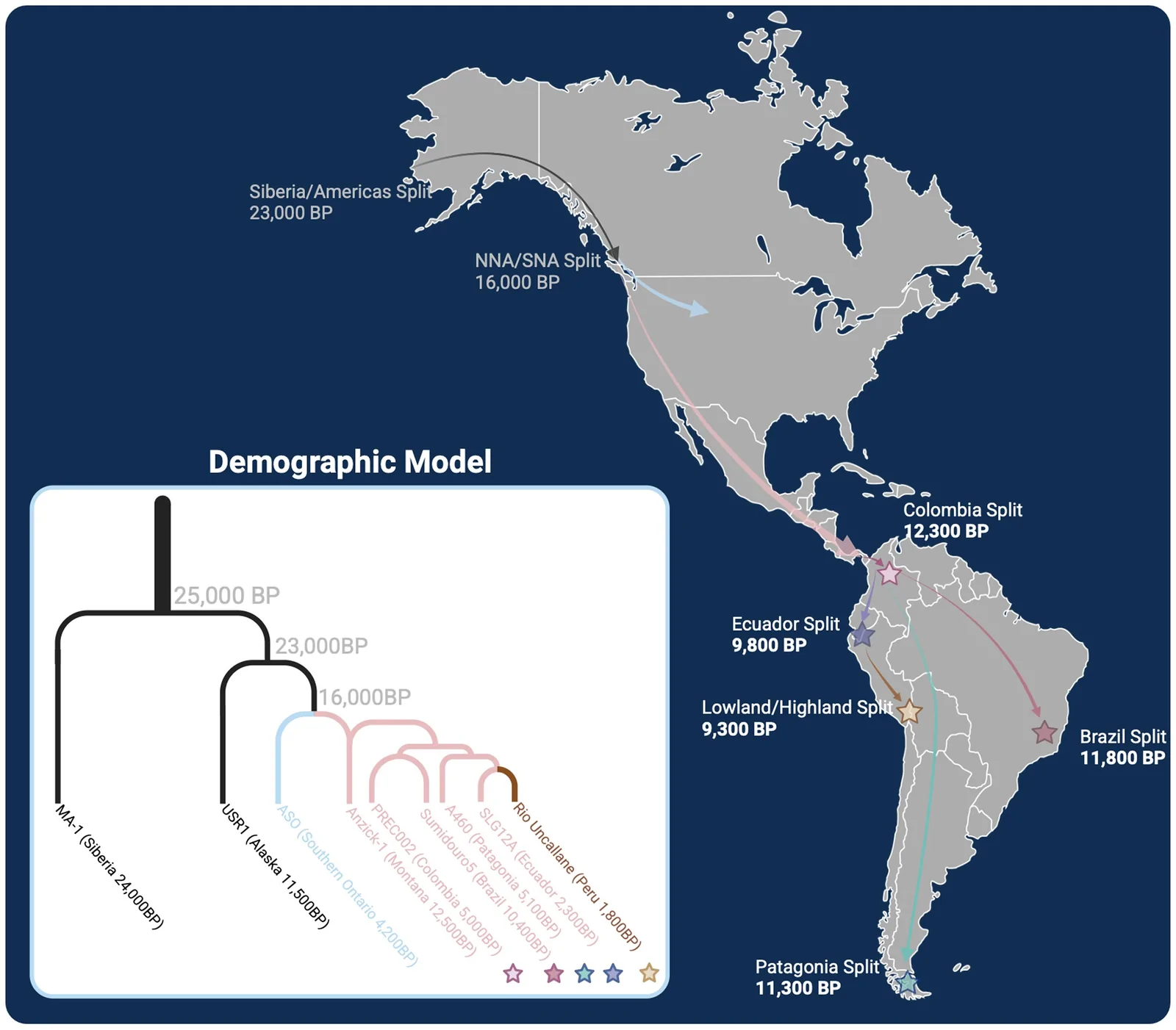
Demographic model and estimated divergence times for the Salango genomes within a continental framework. The best-fitting fastsimcoal2 model was inferred from the joint site-frequency spectrum of ancient and present-day Indigenous populations from North and South America. Gray nodes indicate fixed divergence times (Siberia–Americas, ∼23 ka; Northern Native American–Southern Native American, ∼16 ka), whereas colored nodes indicate median divergence-time estimates from 100 parametric bootstrap estimations. The Salango lineage diverged from Rio Uncallane at 9.30 ka (95% bootstrap interval: 9.18–9.38 ka) and from the inferred ancestral Southern Native American lineage at 9.80 ka (9.63–10.10 ka). The estimated divergences of the Patagonia, Brazil/Sumidouro, and Colombia lineages were 11.33 ka (11.20–11.33 ka), 11.83 ka (11.73–11.88 ka), and 12.30 ka (12.18–12.30 ka), respectively. These intervals are conditional on the specified model topology, fixed deep divergence times, and archaeological calibration.

To evaluate the influence of these assumptions, we conducted additional sensitivity analyses using expanded prior ranges for effective population sizes and South American divergence times, together with alternative values for the fixed temporal anchor (File S2; Figures S15–S17). Under the expanded sequential models, the inferred South American divergence times shifted toward older values as the anchor was increased and frequently clustered near the upper limit permitted by each parameterization. For example, the 5 sequential divergence estimates clustered between 583 and 590 generations under a 600-generation anchor, between 621 and 630 generations under a 640-generation anchor, and between 680 and 688 generations under a 700-generation anchor. A simplified rapid-radiation parameterization likewise supported a temporally compressed diversification. These results indicate that the general signal of closely spaced South American divergences is more consistent across model specifications than either their exact absolute dates or their fine-scale temporal ordering.

Two aspects of the demographic analysis are therefore most informative. First, the constrained model is consistent with demographic differentiation between coastal Ecuador and the central Andes, but the absolute date assigned to that divergence depends appreciably on the archaeological calibration and permitted parameter ranges. Second, the analyses consistently place the Salango lineage within a relatively compressed phase of Southern Native American diversification, broadly agreeing with previous studies proposing rapid population dispersal and the early development of regional population structure in South America (26, 32). The demographic estimates should consequently be viewed as model-dependent temporal scenarios rather than definitive dates for discrete population-splitting events.

Overall, the demographic modeling complements the PCA and outgroup-*f*_3_ and *f*_4_ analyses by providing a formal framework for evaluating the position of the Salango lineage within Southern Native American population history. Although the precise divergence dates remain sensitive to model assumptions and will require additional ancient genomes from coastal Ecuador and northwestern South America to resolve, the results consistently associate Salango with the early diversification of Southern Native American lineages and extend demographic reconstruction to a region previously absent from ancient genomic studies.

### Evidence of *M. tuberculosis* infection in a Guangala infant

Beyond demographic insights, ancient DNA from Salango also sheds light on the disease ecology of Ecuador's early coastal populations. SLG-12B_2300BP derives from infant burial from the Very Early Guangala, previously described as exhibiting skeletal lesions consistent with TB (33). Macroscopic examination revealed lytic lesions and porosity along the inferolateral and lateroposterior edges of both scapulae; plaques of woven bone on the right humerus (and to a lesser extent on the left); and lytic lesions affecting the right first rib, 5 left rib ends, and both ulnar and radial shafts. Additional lytic lesions were present on the left radial tuberosity and the right ilium. The cranium exhibited extensive porotic hyperostosis on the right parietal and superior occipital bones, serpentine endocranial lesions on the temporal and occipital, and porosity across the pars lateralis, basilar portion, and mandible (Figure 5). This constellation of lesions is most consistent with congenitally acquired TB or tuberculous meningitis, likely contracted shortly after birth (34).

**Figure 5:**
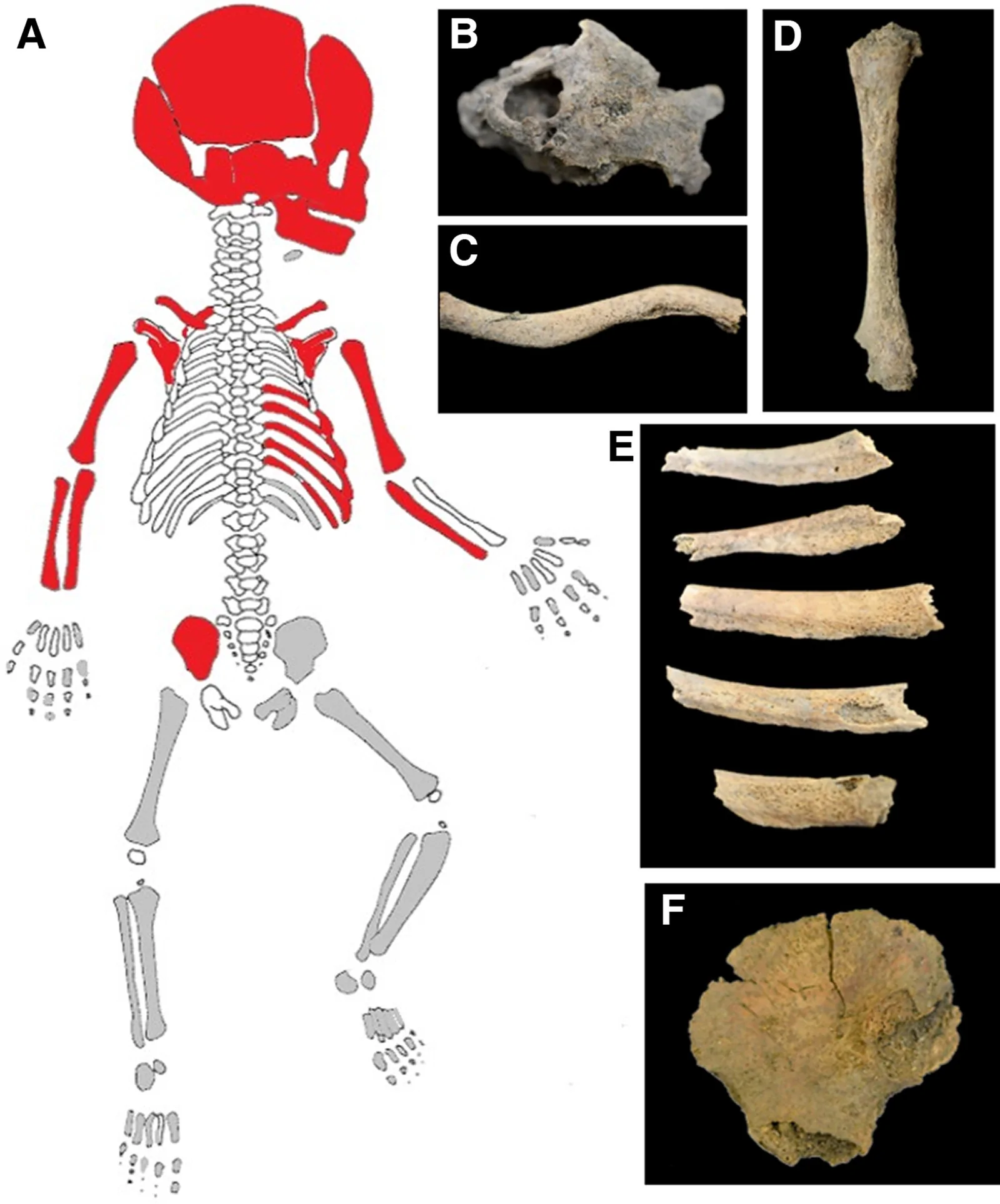
Skeletal elements of Burial 369 (SLG-12B). (A) Showing lesions (red); gray bones were not recovered. (B) Petrous portion with lytic lesions and new bone formation. (C) Left clavicle and (D) right humerus with new bone plaques. (E) Ribs with lytic lesions and (F) right ilium with a large lytic defect.

A left petrous portion showing lytic lesions was sampled for ancient DNA analysis. Metagenomic screening and authentication confirmed the presence of MTBC DNA. Using Kraken2 (35), we identified over 8,000 reads assigned to *M. tuberculosis* (taxID 1773), together with additional reads assigned to closely related MTBC taxa, consistent with the presence of MTBC DNA, including *M. tuberculosis* variant caprae, variant bovis, and *M. canettii*. The Kraken2 profile identified numerous reads assigned to the MTBC, motivating subsequent mapping and authentication analyses (Figure S5).

To further test this finding, we aligned the SLG-12B reads to the *M. tuberculosis* H37Rv reference genome and reconstructed a ∼4× genome, as confirmed by mosdepth (36) coverage profiling (mean depth = 4.65×; max depth = 216×). We then used aRchaic (37) with *k* = 2, including a modern TB control sample for comparison, to assess DNA authenticity via postmortem damage patterns. The SLG-12B TB genome exhibits a cytosine deamination damage (C→T at 5′ ends, G→A at 3′ ends), whereas the modern control lacked such patterns (Figure S3). This suggests that the TB signal in SLG-12B is ancient and endogenous.

Given the moderate genomic coverage of the reconstructed pathogen genome, we refrain from detailed phylogenetic interpretation. Nevertheless, the combined osteological and molecular evidence provides strong support for MTBC infection in this infant and represents one of the earliest molecular confirmations of TB in pre-contact Ecuador. Together with the burial's distinctive mortuary context—interred beneath another juvenile and accompanied by ritual materials—these findings indicate that TB was present among coastal populations of Ecuador by at least ∼2,300 years ago.

## Discussion

Our genomic analyses provide the first direct genetic evidence from ancient Ecuador, filling a major geographic gap in ancient DNA studies of South America and offering new insights into the demographic history of the Pacific coast. Despite substantial environmental disruption following the Pululahua volcanic eruption and major cultural transitions spanning the Very Early Guangala through the Early Manteño periods, the Salango individuals exhibit remarkable genetic continuity over approximately 1,500 years. This long-term persistence is consistent with archaeological evidence indicating enduring occupation, sustained exchange networks, and continuity in ritual and cultural practices at Salango. Together, these findings suggest that cultural transformations along Ecuador's central coast occurred largely within a stable local population rather than through large-scale population replacement.

Patterns of shared genetic drift and allele sharing place the Salango individuals within a broad Southern Native American genetic continuum while revealing changing regional connections through time. The Very Early Guangala individuals show shared drift with a geographically and temporally diverse set of populations, including coastal Peru, Andean populations, early Holocene genomes from Brazil and the Lake Titicaca region, and selected populations from the Pacific coast and adjacent regions. The Early Manteño individual SLG-27B retains these broader affinities but shows relatively stronger connections with later Andean and western South American populations, including individuals from Bolivia, Chile, Peru, and the Ecuadorian highlands. Direct *f*_4_ comparisons among the Salango individuals further support substantially greater allele sharing among them than with comparative populations, consistent with demographic continuity across the Guangala and Manteño phases. Together, these results support long-term continuity at Salango accompanied by changing patterns of coastal–highland interaction and regional connectivity, rather than population replacement.

Our demographic modeling provides a model-based framework for placing the Salango lineage within the early diversification of Southern Native American populations. The consistent qualitative result across the demographic analyses is a temporally compressed period of differentiation among the sampled Southern Native American lineages. This pattern is broadly compatible with rapid population dispersal and the development of regional population structure following the initial settlement of South America, although the available data do not uniquely determine the timing or exact sequence of the individual divergences. The relationship inferred between Salango and Rio Uncallane is consistent with demographic differentiation between coastal Ecuador and the central Andes, while the allele-sharing analyses indicate that these regions participated in changing networks of interaction through time. Additional ancient genomes from coastal Ecuador and northwestern South America will be essential for testing whether the pattern observed at Salango was geographically widespread and for obtaining less model-dependent estimates of the timing of regional population differentiation.

In addition to these demographic insights, the recovery of authenticated MTBC DNA from the infant SLG-12B provides molecular support for the osteological diagnosis of TB. The characteristic ancient DNA damage patterns and genomic reconstruction confirm that MTBC infection was present among coastal Ecuadorian populations by at least ∼2,300 years before present. While the moderate genomic coverage of the reconstructed pathogen genome limits detailed phylogenetic interpretation, the combined osteological and molecular evidence expands the growing record of TB in pre-contact South America and demonstrates the potential for recovering ancient pathogen genomes from archaeological remains in Ecuador. Future analyses of additional ancient MTBC genomes from the region will be necessary to better understand the evolutionary history and geographic diversity of TB before European contact.

Together, these findings demonstrate that Ecuador's Pacific coast was an enduring center of human occupation that maintained long-term genetic continuity while participating in broader networks of interaction linking coastal and Andean populations. By providing the first ancient genomes from Ecuador, this study provides a valuable genomic reference for future investigations of population history, mobility, and adaptation in northwestern South America and underscores the importance of including previously unsampled regions in continental reconstructions of the peopling of the Americas.

### Ethics statement

All research was conducted in full compliance with institutional and national ethical guidelines governing the study of ancient human remains. Ancient DNA sampling and analysis were approved by the Emory University Institutional Review Board (IRB #IRB00104677) and adhered to best practices for ancient DNA research, including minimal destructive sampling, secure storage of extracted material, and secured data management.

All excavation, sampling, and export of archaeological materials were authorized by Instituto Nacional de Patrimonio Cultural (Memorando N° INPC-PI-R4-2018-0111-M). During the archaeological expedition, Richard Lunniss engaged with members of the local community throughout the excavation. The genomic analysis of samples was authorized by the *Comunidad Ancestral Salango*—the legally recognized descendant community of the Salango archaeological site—under formal community consent (Oficio N° CAS-00323, October 2, 2024). The community assembly and governing council explicitly endorsed the project on the conditions that (i) all results be shared with the community and the *Centro de Investigaciones y Museo Salango*; (ii) all ancestral remains be repatriated and permanently curated at the Salango Museum; and (iii) a joint protocol be established to govern the export and future study of prehistoric materials. Coauthor Daniel Rivas Álava will collaborate directly with the *Comunidad Ancestral Salango* to draft and implement this protocol to ensure transparent, equitable, and culturally appropriate oversight of ongoing and future research. A full copy of the community's authorization and letter of support is included in Note S1.

## Methods

### Archeological context

The sources of the samples were recovered during rescue excavations supported by the Universidad Técnica de Manabí and carried out at Salango by Richard Lunniss and a team of comuna field archaeologists between December 2014 and January 2016 (38). Sample SLG-9B and Samples SLG12A and SLG12B came from stratigraphic excavation of a burial mound at Calle 22, near the north edge of the sanctuary in its Very Early Guangala phase. Sample SLG-9B belonged to Context 339, an infant of 6–9 months who was buried wearing, over the head, the modified portion of another infant skull (13). Samples SLG12A and SLG12B came from Context 369, an infant also of 6–9 months but with an enlarged cranium and skeletal lesioning both suggestive of TB (13). Sample SLG-33B came the secondary urn burial of an infant (between birth and 6 months) of the Early Guangala phase excavated in Calle Larga after partial exposure by machine excavation. Sample SLG-27B was collected from an adult Late Manteño burial (between 25 and 40 years of age) also exposed by machine digging in Calle Larga. The Early Guangala origin of Sample SLG-33B is indicated by burial configuration and associated pottery. The cultural origin of Sample SLG-27B is based solely on radiocarbon dating.

### Ancient DNA sampling, extraction, and library preparation

All pre-PCR work was performed in the dedicated clean facilities of the Lindo Ancient DNA Laboratory (Emory University) following strict contamination-control procedures. From each individual, either a tooth or petrous bone was utilized for the extraction. The teeth were surface-decontaminated by brief immersion in 6–10% sodium hypochlorite, rinsed in molecular-grade water, air-dried in a UV cabinet, and abraded to remove the outer layer. ∼30–100 mg of internal powder was generated using a sterile dental bur. DNA was extracted using the silica-based method of Dabney et al. (39). Briefly, bone/tooth powder was decalcified in 0.45M EDTA (pH 8.0) with Proteinase K (0.25–0.5 mg/mL) at 37°C with rotation (overnight), supernatants were cleared by centrifugation, and DNA was bound to silica in guanidinium-based binding buffer with isopropanol. Silica columns were washed with 80% ethanol, and DNA was eluted in 50 µL TET (10 mM Tris-HCl pH 8.0, 1 mM EDTA, 0.05% Tween-20). Extraction blanks were carried forward to library construction and sequencing.

Sequencing libraries were prepared from 10 to 25 µL of extract using the ClaretBio SRSLY PicoPlus Uracil + kit, following the manufacturer's “Uracil+” workflow optimized for damaged/uracil-containing ancient templates and low input DNA. The SRSLY protocol builds single-stranded–compatible libraries via adapter ligation, followed by limited-cycle PCR to add unique dual indexes. Library amplification cycle numbers were determined by qPCR to avoid over-amplification; amplification used the Uracil-tolerant polymerase supplied/recommended with the kit. Post-PCR libraries were purified with SPRI beads (1.2×) and assessed by Qubit fluorometry and fragment analysis. Library blanks accompanied each batch.

Unless otherwise noted, no additional UDG treatment beyond the kit's Uracil+ workflow was applied, preserving terminal damage signals used for downstream authentication. Indexed libraries (including blanks) were pooled equimolarly for Illumina sequencing. Downstream authentication metrics (fragment length distributions, 5′-C→T/3′-G→A misincorporations, and contamination estimates) are provided in Figure S1 and Table 1.

### Bioinformatics processing and data analysis

Raw sequencing reads were processed using standard ancient DNA (aDNA) bioinformatics workflows optimized for short, damaged fragments. Adapter sequences were trimmed and reads shorter than 30 bp were removed prior to alignment. Filtered reads were aligned to the human reference genome (hg19) using BWA (40) aln (v0.7.17) with parameters recommended for aDNA (-l 1024 -n 0.01 -o 2), which disable seeding and increase tolerance for mismatches associated with postmortem damage. Aligned reads were converted to BAM format and sorted using SAMtools (41) (v1.19). PCR duplicates were identified and removed with PicardTools MarkDuplicates to ensure independent fragment representation. Alignment summary statistics and per-base coverage were calculated using mosdepth (v0.3.3). Because all genomes were sequenced at low coverage (0.5–2.3×), we generated pseudo-haploid calls by randomly sampling one high-quality base per site using a custom script, minimizing bias from differential coverage and DNA damage. These pseudo-haploid calls were merged across individuals using bcftools merge (v1.17), together with reference data from the Simons Genome Diversity Project (42) (SGDP) and previously published ancient genomes from the Americas and Siberia (Table S2). All downstream analyses—including PCA, outgroup *f_3_* and *f_4_* statistics, and demographic modeling—were conducted using the merged pseudo-haploid dataset. Only sites covered in at least 95% of reference individuals were retained to reduce missing-data artifacts. Transitions (C↔T, G↔A) were excluded to minimize potential impacts of postmortem deamination.

### Principal component analysis

To investigate the genetic affinities of the Salango individuals within a broader continental context, we performed PCA using *smartpca* from the EIGENSOFT package (v7.21). The primary analysis, presented in the main manuscript, included ancient and present-day Indigenous American individuals together with northern North American and Siberian reference populations (Table S2). Principal component axes were calculated using high-coverage present-day Indigenous American individuals, while ancient individuals, including those from Salango, were projected onto these axes using the parameters lsqproject: YES and shrinkmode: YES. These settings implement least-squares projection and shrinkage correction, respectively, thereby preventing individuals with sparse genotype data from influencing the eigenvector space while allowing their genetic affinities to be visualized relative to present-day population structure. The primary analysis included 54,403 polymorphic sites.

To further examine regional population structure and address the possibility that the broader composition of the dataset obscured differentiation among Central and South American populations, we conducted a second PCA restricted to Indigenous American comparative populations (Figure 2). This expanded regional analysis incorporated 4 ancient coastal Peruvian individuals selected from the Allen Ancient DNA Resource: I2241 from El Brujo, Lambayeque (∼925 BP); I2262 from El Brujo, associated with the Moche cultural context (∼1550 BP); I0966 from Huaca Pucllana, Lima, associated with the Ychsma cultural context (∼750 BP); and UC8_8173 from the Chincha Valley (∼533 BP). These individuals were selected to improve temporal and geographic representation of the Peruvian coast while maximizing genotype coverage and the number of sites overlapping the Salango dataset. For the regional PCA, the axes were defined by 10 present-day Indigenous individuals representing Aymara, Quechua, Karitiana, Surui, Maya, and Piapoco populations. Ancient individuals and other regional comparative individuals were projected using lsqproject: YES and shrinkmode: YES. Variants with more than 50% missing data across the combined dataset were excluded, followed by a minor-allele-frequency threshold of 0.05 and linkage-disequilibrium pruning using a 200-kb window, a one-variant step, and an *r*^2^ threshold of 0.2. The resulting regional PCA included 49,211 LD-pruned SNPs and 62 individuals. PC1–PC2 and PC3–PC4 are presented at both the complete regional scale and at an expanded scale restricted to ancient Central and South American individuals. All 4 retained Salango individuals—SLG-9B, SLG-12B, SLG-27B, and SLG-33B—were projected in this analysis. Because only 664 of the PCA SNPs were available for SLG-33B, its projected position was considered unstable and was not interpreted as evidence of population differentiation.

### Outgroup *f*_3_ and *f*_4_-statistics

To formally assess shared genetic drift and test for asymmetry in allele frequency covariance, we computed outgroup *f*_3_ and *f*_4_ statistics using the ADMIXTOOLS2 R package. Analyses were performed on pseudo-haploid genotype data derived from the merged dataset of ancient and present-day Indigenous American individuals (Table S2). Depending on overlap among samples, a range of 124,691 to 341,116 polymorphic sites were included per test.

Outgroup *f_3_* statistics of the form *f_3_*(Mbuti; X, SLG) were used to quantify the amount of shared genetic drift between each Salango individual and comparison populations since their divergence from the African outgroup Mbuti. These values provide a robust measure of shared ancestry, largely insensitive to postmortem DNA damage or sequencing error, and are particularly suited for low-coverage ancient genomes.

To examine potential deviations from a strictly tree-like relationship and to detect differential allele sharing, we computed *f_4_* statistics of the form *f_4_*(Mbuti, SLG; Athabaskan, X), where Athabaskan was used as a post–Northern Native American (NNA) reference population. Positive or negative *f_4_* values indicate asymmetry in allele frequency covariance, reflecting excess genetic affinity of SLG with population X relative to the NNA lineage. Standard errors were estimated via block jackknifing across 5 Mb genomic blocks to account for linkage disequilibrium. Results were visualized using custom R scripts, with |Z| ≥ 3 considered statistically significant. Complete outgroup-(f_3) results, including the number of overlapping SNPs, estimates, block-jackknife standard errors, and Z-scores, are provided in Table S3. Of note, the SMP5 genotypes used in our analysis were not obtained from the Allen Ancient DNA Data Resource. Instead, pseudo-haploid genotypes were generated directly from the original shotgun-sequencing BAM file.

### Demographic inference using fastsimcoal2

To investigate the demographic history of the Salango population from coastal Ecuador in relation to other ancient populations from the Americas, we performed demographic inference with fastsimcoal2 v2.8 using a multidimensional joint site-frequency spectrum (SFS). The primary analysis included 9 populations: Russia (MA-1, 24,000 BP), Alaska (USR1, 11,400 BP), Canada (Ontario, 2,895 BP), Brazil (Sumidouro5, 10,400 BP), Colombia (5,000 BP), Patagonia, Rio Uncallane, Salango (SLG), and Anzick-1. The derived joint SFS was generated using easySFS (43), with each population projected to 2 chromosomes to balance retention of informative segregating sites against missing data. Pairwise 2-dimensional SFS and marginal SFS are presented in Figures S13 and S14. The primary demographic model specified a sequential series of population-merger events backward in time. Rio Uncallane first merged with Salango, followed by the merger of the Salango lineage with the broader Southern Native American lineage and then sequential mergers involving Patagonia, Brazil/Sumidouro, and Colombia. Deeper events were fixed at 640 generations for the Anzick–Ontario divergence, 785 generations for the Ontario–USR1 divergence, and 892 generations for the USR1–MA-1 divergence. An ancestral population-size change to 3,000 haploid genomes was fixed at 1,785 generations. Effective population sizes were estimated independently. The stated uniform prior ranges were 500–12,000 haploid genomes for Ontario, Sumidouro, Salango, and Anzick; 500–16,000 for Colombia and Patagonia; and 500–20,000 for Rio Uncallane. The archaeology-constrained divergence-time priors were 360–379 generations for the Rio Uncallane–Salango divergence, 380–440 generations for the divergence of Salango from the broader Southern Native American lineage, 441–460 generations for Patagonia, 461–480 generations for Brazil/Sumidouro, and 481–500 generations for Colombia. These ranges were originally intended to impose the hypothesized temporal ordering of the South American divergences within an archaeologically plausible interval. Because several ranges were narrow, particularly the approximately 20-generation intervals, we subsequently evaluated their influence through expanded-prior and anchor-sensitivity analyses described below. A mutation rate of 1.25 × 10^−8^ substitutions per site per generation, a recombination rate of 2 × 10^−8^ per site per generation, and a generation time of 25 years were assumed. Each primary-model optimization used 100,000 coalescent simulations per likelihood-estimation cycle and 40 expectation–conditional maximization cycles. We conducted 100 independent optimizations using different random seeds. The observed-data parameter set with the highest composite likelihood was retained as the primary maximum-likelihood solution. Maximum observed and estimated composite likelihoods were recorded as optimization diagnostics but were not interpreted as conventional goodness-of-fit statistics.

#### Expanded-prior and anchor-sensitivity analyses

To evaluate whether the inferred divergence times were driven by the narrow primary-model priors, we fitted expanded sequential models in which all effective population sizes were assigned log-uniform priors ranging from 100 to 100,000 haploid genomes. The South American divergence times were allowed to vary across a shared, substantially broader interval, subject only to the temporal ordering required by the demographic topology. The youngest divergence had a lower bound of 360 generations, and each subsequent divergence was constrained to be no younger than the preceding event. We examined 3 alternative temporal-anchor configurations. In the first, the Anzick–Ontario event was fixed at 600 generations and South American divergence times were permitted to extend to 590 generations. In the second, the anchor was fixed at 640 generations and the upper limit was 630 generations. In the third, the anchor was fixed at 700 generations and the upper limit was 690 generations. The deeper Ontario–USR1 and USR1–MA-1 events remained fixed at 785 and 892 generations, respectively. We also evaluated a reduced rapid-radiation model using the 600-generation anchor. In this model, the Rio Uncallane–Salango divergence was estimated separately, whereas the divergences involving Salango, Patagonia, Brazil/Sumidouro, and Colombia shared a single South American radiation-time parameter. This model contained 9 free parameters, compared with 12 in the expanded sequential models. The sensitivity models were evaluated using a common folded multidimensional SFS configuration constructed from the same 9-population panel. For each model, we conducted 20 screening optimizations using 50,000 coalescent simulations and 20 optimization cycles. The 3 best valid screening solutions were then refined using 200,000 simulations and 50 optimization cycles.

The sensitivity models shared the same observed-likelihood configuration and could therefore be compared with one another using their maximum composite likelihoods and composite Akaike information criterion values. The sensitivity analysis was used to assess the dependence of parameter estimates on the prior limits, temporal anchors, and representation of the South American radiation. Complete sensitivity-model definitions, optimization results, and .tpl and .est files are provided in File S2.

#### Parametric bootstrap estimation of uncertainty

Parameter uncertainty for the primary model was evaluated using a conditional parametric bootstrap procedure. First, an expected multidimensional SFS was generated at the original maximum-likelihood parameter set using 1,000,000 coalescent simulations. We then generated 100 pseudo-observed SFS replicates by multinomial sampling from the expected SFS, conditional on the observed total of 388,673 polymorphic sites. Each bootstrap replicate was independently re-estimated under the primary demographic topology using the explicitly bounded working.est file. Re-estimation used 100,000 coalescent simulations, and 40 expectation–conditional maximization cycles. Empirical 95% bootstrap intervals were calculated as the 2.5th and 97.5th percentiles of the 100 re-estimated parameter values. The number of estimates reaching either prior boundary was also recorded.

These intervals incorporate uncertainty arising from multinomial sampling of the SFS, coalescent simulation, and numerical optimization under the specified model. They remain conditional on the demographic topology, fixed divergence times, mutation and generation-time calibrations, SFS construction, and parameter bounds. Figure S15 compares the stated priors with distributions of independently optimized and bootstrap-re-estimated parameters. Additional supplementary figures show the response of the divergence-time estimates to alternative temporal anchors and the optimization results for the sensitivity models (Figures S16 and S17).

### Identification and authentication of *M. tuberculosis*

Non-human reads from individual SLG-12B were taxonomically classified using Kraken2 (v2.1.2) with the standard Kraken2 PlusPF database, revealing a high-confidence signal for the MTBC, including 8,367 reads assigned directly to *M. tuberculosis* (taxID: 1773) and additional reads mapping to related MTBC lineages and environmental *Mycobacterium* species. Following taxonomic screening, we aligned SLG-12B sequencing reads to the *M. tuberculosis* H37Rv reference genome (NC_000962.3) using BWA aln (v0.7.17) with ancient-DNA-optimized parameters (-n 0.01 -o 2). PCR duplicates were removed using samtools markdup, and depth of coverage was assessed using mosdepth (v0.3.1), revealing an average coverage of 4.65×, with a maximum depth of 216×, across the 4.41 Mb *M. tuberculosis* genome.

To authenticate the ancient origin of the recovered sequences, we analyzed damage patterns using aRchaic (v1.0.2), specifying *k* = 2 and including a modern *M. tuberculosis* genome from the SRA (accession: *ERS23402117*) as a negative control. The SLG-12B profile exhibited elevated C→T misincorporations at 5′ read ends and G→A at 3′ ends, a hallmark of postmortem cytosine deamination in ancient DNA. In contrast, the modern control showed no such terminal damage, confirming the authenticity of the ancient TB signal in SLG-12B.

To ensure taxonomic specificity, we implemented a 2-tier contaminant filtering strategy using Kraken2. After initial alignment of SLG-12B reads to the TB reference genome, we converted the BAM file to FASTQ and reclassified all reads using Kraken2 with a comprehensive microbial database. Sequences not assigned to the MTBC were filtered out and removed from the BAM file before downstream consensus generation and phylogenetic alignment. This approach minimizes spurious placement of ancient sequences due to non-MTBC environmental contaminants.

### Bioarchaeological identification of TB

Juengst macroscopically analyzed Burial 339 at the Universidad Técnica de Manabí in 2018 with the assistance of Abigail Bythell and several UTM undergraduate students. This analysis included age estimation and observation of paleopathological indicators including cribra orbitalia, porotic hyperostosis, linear enamel hypoplasia, lytic and sclerotic reactions, osteomyelitis, and the presence of auditory exostoses. Age-at-death was estimated based on tooth eruption and tooth root formation (44). Porotic cranial lesions were recorded by location and size of defect following standard methods in Buikstra and Ubelaker (45). Linear enamel hypoplasias were recorded as present or absent and counted in frequency per tooth and per individual (46, 47). Juengst recorded lytic and sclerotic reactions and osteomyelitis by location, skeletal element, and extent of the lesion. Reactions were identified by new bone formation, hypervascularity, and appositional bone deposits. Healing was recorded as fully healed (woven bone was remodeled although defect was still notable), some healing (woven bone was partially smoothed but active areas were still observable), and active (no evidence for healing) (45, 48). We additionally recorded osteomyelitis by obstruction of the marrow cavity (if observable) and the presence of draining sinuses or cloaca (45, 48). Full paleopathological results for these analyses are presented in Juengst et al. (48).

## Supplementary Material

pgag323_Supplementary_Data

## Data Availability

All newly generated genome-wide sequence data from the Salango individuals are publicly available through the NCBI Sequence Read Archive (SRA) under BioProject accession PRJNA1357898.
