# Supplementary material for: Ancient Ecuadorian genomes reveal millennia of genetic continuity on the Pacific Coast of South America
Source: PNAS Nexus. 2026 Oct 27;5(10):pgag323. doi: 10.1093/pnasnexus/pgag323 (PMC13711318; doi:10.1093/pnasnexus/pgag323)
Supplement: Supplementary file 1 [file pgag323_supplementary_data.zip › PNASNEXUS-PNASNEXUS-2026-01334R-s01.docx]

**Supplement**

**Ancient Ecuadorian Genomes Reveal Millennia of Genetic Continuity on the Pacific Coast of South America**

John Lindo^1*^, Daniel Rivas Alava^2,^ Sara Juengst^3^, Yemko Pryor^4^, Sophie K. Joseph^5^, J.J. Ortiz Aguilu^6^, Richard Lunniss^7*^

^1^Department of Anthropology, University of California, Berkeley CA, 97420 USA

^2^Department of Anthropology, Emory University, Atlanta GA, 30322 USA

^3^Department of Anthropology, University of North Carolina at Charlotte, Charlotte, NC 28223 USA

^4^Department of Anthropology, University of Michigan, Ann Arbor MI, 48109 USA

^5^Department of Molecular and Cell Biology, University of California, Berkeley, Berkeley, CA 94720 USA

^6^Üniversidad Interamericana De Puerto Rico- Recinto De Barranquitas, Barranquitas, Puerto Rico, 00794

^7^Universidad Tecnica de Manabı´, 130105 Manabı, Ecuador

*Corresponding authors: jlindo@emory.edu; richard.marshall@utm.edu.ec

**Table of Contents**

Figure S1. DNA Damage Patterns	3

Figure S2. Regional PCA of Indigenous populations from the Americas	4

Figure S2: Affinity and allele-sharing patterns for SLG-12B	4

Figure S3: Unsupervised Neural ADMIXTURE analysis	5

Figure S4: Outgroup-*f*3 affinities between SLG-27B and SLG-27B	6

Figure S5: Genetic affinities and allele-sharing patterns for SLG-12B	7

Figure S6. Non-Athabascan temporal *f*4 contrasts at Salango	8

Figure S7. Highland–coastal temporal f4 tests using individual Salango samples	9

Figure S8. Highland–coastal temporal *f*4 tests using pooled early Salango individuals	10

Figure S9. Sumidouro5-specificity tests using individual Salango samples	11

Figure S10. Sumidouro5-specificity tests using pooled Early Salango	12

Figure S11. Deamination damage profile for ancient TB recovered from individual SLG-12B	13

Figure S12. Kraken2 taxonomic assignments of *Mycobacterium tuberculosis* complex reads from SLG-12B	13

Figure S13. Folded two-dimensional site frequency spectra comparing ancient Salango individuals	with other ancient South American populations	14

Figure S14. One-dimensional marginal site frequency spectra for populations included in the fastsimcoal2 demographic model.	15

Figure S15. Parameter constraints and empirical estimate distributions for FastSimCoal2 model	16

Figure S16. Sensitivity of sequential South American divergence-time estimates	17

Figure S17. Optimization diagnostics for the final sensitivity models	18

Table S1. Published samples used in this study	19

Table S2: Radiocarbon dating report from the UGA Center for Applied Isotope Studies	20

Table S3. Parameter estimates from the fastsimcoal2 demographic model	21

Supplementary Note 1: Fastsimcoal2 tpl file used for demographic simulation	22

Supplementary Note 2: Fastsimcoal2 est file used for demographic simulation	23

Supplementary Note 3. Research agreement with the Comunidad Ancestral Salango	24

Supplementary References

25

**Supplementary File 1: *f*_3_ & *f*_4_ Statistics**

**Supplementary File 2: Fastsimcoal2 Parameters, Outputs and Bootstrapping for Sensitivity Models**

**Figure S1.** **DNA damage patterns of reads recovered from Salango individuals.** Deamination profiles show characteristic cytosine-to-thymine (C→T) misincorporations at the 5′ ends (blue) and guanine-to-adenine (G→A) at the 3′ ends (red), consistent with ancient DNA. Each panel represents an individual sample (SLG-9B, SLG-12B, SLG-27B, and SLG-33B).

**Figure S2.** **Regional PCA of Indigenous populations from the Americas.** Principal components were calculated using 10 present-day Indigenous reference individuals from the Aymara, Quechua, Karitiana, Suruí, Mayan, and Piapoco populations based on 49,211 LD-pruned SNPs. Ancient individuals and other regional comparative individuals were projected using least-squares projection with shrinkage correction. The analysis includes four ancient Peruvian coastal individuals from Lambayeque, Moche, Lima–Ychsma, and the Chincha Valley. (A) Full PC1–PC2 coordinate range, including the present-day reference populations, comparative individuals, and the Salango individuals. (B) Expanded view of the projected ancient individuals on PC1–PC2. (C) Full PC3–PC4 coordinate range. (D) Expanded view of the projected ancient individuals on PC3–PC4. The expanded panels retain the original PCA coordinates and differ only in the displayed axis ranges. Colors and symbols distinguish the Salango individuals, ancient Peruvian coastal individuals, comparative ancient populations, and broad geographic groups. Although SLG-33B is displayed for completeness, only 664 SNPs were available for its projection. Its position should therefore be interpreted cautiously. The percentage of reference-panel variation explained by each principal component is shown on the corresponding axis.

**Figure S3.** **Unsupervised Neural ADMIXTURE analysis.** Genetic structure was assessed using Neural ADMIXTURE v1.6.1 in unsupervised mode across K = 2–8 ancestral components. Variants were restricted to biallelic sites and filtered in PLINK using --max-alleles 2 --mac 1 --maf 0.05; linkage disequilibrium pruning was performed with --indep-pairwise 1000 100 0.2. We retained only sites with genotype calls for every included individual, yielding a complete matrix of 27,681 LD-pruned autosomal SNPs with no missing data among 62 individuals. Each vertical bar represents an individual; colors denote model components and are arbitrary across K values. The three Salango individuals retained after complete-data filtering (SLG-12B, SLG-27B, and SLG-9B) consistently form a near-exclusive component across K values, indicating genetic cohesion and differentiation relative to the included comparison panel. Cluster assignments were visualized using pong(1). Because model-based clustering components may also capture post-divergence drift, reference-panel composition, and characteristics of low-coverage pseudo-haploid ancient data, they are interpreted descriptively alongside PCA and f-statistics rather than as direct estimates of discrete ancestral populations or admixture proportions.

**Figure S4.** **Outgroup-*f*3 affinities between SLG-27B and SLG-27B.** Higher f3 values indicate greater shared genetic drift. (A) Results plotted using the complete f3 range, including SLG-9B. The SLG-27B–SLG-9B comparison produced the highest value (f3 = 0.717). (B) The color scale is expanded across the non-Salango comparisons (f3 = 0.097–0.150) to show geographic variation that is obscured by the much higher within-Salango value. SLG-9B is retained as a black diamond and identified as being above the displayed color scale.

**Figure S5. Genetic affinities and allele-sharing patterns for SLG-12B (~2300 BP).** (A) Outgroup-f3 statistics of the form f3(Mbuti; X, SLG-12B), where X represents each comparative population. Populations are ranked by decreasing f3 estimate. Points indicate f3 estimates, horizontal bars indicate uncertainty, and point colors correspond to the f3 estimate shown in the color scale. Higher f3 values indicate greater shared genetic drift between SLG-12B and the comparison population relative to the Mbuti outgroup. (B) f4 statistics of the form f4(Mbuti, SLG-12B; Athabascan, X). Points indicate f4 estimates and horizontal bars indicate 95% confidence intervals. Positive values indicate greater allele sharing between SLG-12B and the comparison population X relative to Athabascan, whereas negative values indicate comparatively greater allele sharing between SLG-12B and Athabascan. Upward triangles indicate significant positive values (Z > 3), circles indicate nonsignificant values, and downward triangles indicate significant negative values (Z < −3). The number of overlapping SNPs is provided for each comparison.

**Figure S6. Non-Athabascan temporal *f*4 contrasts at Salango.** Each panel shows comparisons of the form f4(Mbuti, X; Salango individual 1, Salango individual 2), where X is the indicated South or Central American reference population. Panels correspond to the SLG-12B versus SLG-27B, SLG-9B versus SLG-12B, and SLG-9B versus SLG-27B contrasts. Points indicate f4 estimates and horizontal bars indicate 95% confidence intervals. Positive and negative values represent asymmetrical allele sharing between the two Salango individuals relative to X. Populations are ordered by the f4 estimate. No comparison reaches |Z| > 3.

**Figure S7.** **Highland–coastal temporal f4 tests using individual Salango samples.** Statistics have the form f4(early Salango, SLG-27B; highland reference, coastal reference). The panels show tests for SLG-9B and SLG-12B, respectively. Positive values favor the early-Salango/highland and SLG-27B/coastal configuration; negative values favor the reciprocal configuration. Points indicate estimates and bars indicate 95% confidence intervals. No comparison reaches |Z| > 3.

**Figure S8. Highland–coastal temporal *f*4 tests using pooled early Salango individuals.** Statistics have the form f4(Early Salango, SLG-27B; highland reference, coastal reference). Positive values favor the Early Salango/highland and SLG-27B/coastal configuration; negative values favor the reciprocal configuration. Points indicate estimates and bars indicate 95% confidence intervals. No comparison reaches |Z| > 3.

**Figure S9. Sumidouro5-specificity tests using individual Salango samples.** Statistics have the form f4(Mbuti, Salango individual; Brazil_Sumidouro5, alternative reference). Negative values indicate greater allele sharing between the Salango individual and Brazil_Sumidouro5 relative to the alternative reference; positive values favor the alternative reference. Panels show SLG-9B, SLG-12B, and SLG-27B. Points indicate estimates and bars indicate 95% confidence intervals.

**Figure S10. Sumidouro5-specificity tests using pooled Early Salango.** Statistics have the form f4(Mbuti, Early Salango; Brazil_Sumidouro5, alternative reference). Negative values indicate greater allele sharing between Early Salango and Brazil_Sumidouro5 relative to the alternative reference. Points indicate estimates and bars indicate 95% confidence intervals. Populations are ordered by the f4 estimate.

**Figure S11. *Deamination damage profile for ancient Mycobacterium tuberculosis DNA recovered from individual SLG-12B, modeled using aRchaic with k = 2****.* Misincorporation frequencies show cytosine deamination damage patterns characteristic of ancient DNA. C→T substitutions (red) at the 5′ end and G→A substitutions (blue, mirrored at the 3′ end) are elevated at terminal positions, especially position 0. These patterns support the authenticity of the SLG-12B M. tuberculosis sequence and indicate postmortem cytosine deamination. For comparison, a modern M. tuberculosis genome (SRA accession: *ERS23402117*) was included in the aRchaic mixture model to help differentiate authentic ancient signal from background sequencing error.

**Figure S12. Kraken2 taxonomic assignments of *Mycobacterium tuberculosis* complex reads from SLG-12B.** Bars represent the number of reads assigned to each taxon within the MTBC. Most reads cluster within *M. tuberculosis* and closely related complex members (*M. bovis*, *M. caprae*, *M. canettii*), reflecting conserved genomic regions shared across the complex rather than mixed infection or contamination.

**Figure S13. Folded two-dimensional site frequency spectra comparing ancient Salango individuals with other ancient South American populations.** Each panel shows the joint distribution of derived allele counts between the Salango individuals and a comparison population, providing the summary statistics used for demographic modeling. Similarities and differences among the spectra reflect variation in shared allele frequencies among populations but should not be interpreted independently of the demographic model. The colour scale indicates log₁₀(count + 1) of observed SNP bins.

**Figure S14. One-dimensional marginal site frequency spectra for populations included in the fastsimcoal2 demographic model.** Each histogram shows the distribution of derived allele counts for the populations included in the demographic analyses, including MA1, USR1, Ontario, Sumidouro, Colombia, Patagonia, Rio Uncallane, and Salango (SLG). As expected for ancient genomic datasets, the spectra are dominated by singleton and low-frequency derived alleles. The SLG spectrum exhibits an allele frequency distribution broadly comparable to those of the other South American populations included in the demographic analyses. These marginal spectra provide descriptive summaries of the allele frequency data used to construct the joint site frequency spectrum for demographic inference.

**Figure S15. Parameter constraints and empirical estimate distributions for FastSimCoal2 model.** Shaded regions and dashed lines show the stated lower and upper bounds of the original constrained model. Violin and box plots show estimates from 100 independent optimizations of the observed SFS and 100 parametric-bootstrap re-estimations. Gold diamonds denote the best original maximum-likelihood estimate. Population-size estimates from the legacy optimization occasionally extend beyond the stated ranges because the legacy estimation file did not enforce the bounds under the current fastsimcoal28 syntax. Bootstrap re-estimations explicitly enforced them.

**Figure S16. Sensitivity of sequential South American divergence-time estimates to the fixed temporal anchor.** (A) Best high-resolution estimates from models with anchors of 600, 640, and 700 generations. The grey region shows the permitted time interval and the dashed line its upper bound, set ten generations younger than the anchor. The secondary axis assumes 25 years per generation. (B) Distance between each estimate and its permitted upper bound. Estimates move older with the anchor and remain close to the upper limit, demonstrating that absolute dates are calibration-dependent.

**Figure S17. Optimization diagnostics for the final sensitivity models.** (A) Maximum estimated composite log-likelihoods from independent lower-resolution screening optimizations; gold points mark the best screening result for each model. One nonconvergent rapid-anchor-600 screening run was excluded. (B) Maximum estimated composite log-likelihoods from the three high-resolution refinements selected for each model. Gold points mark the best refined result. Likelihoods are shown separately by optimization phase because the phases used different simulation effort.

| **Individual ID** | **Location** | **Ancient or contemporary** | **Reference** |
| --- | --- | --- | --- |
| Anzick-1 | Montana, USA | ancient | Rasmussen et al. 2014(2) |
| USR-1 | Alaska, USA | ancient | Moreno-Mayar et al. 2018(3) , *Nature* |
| BigBar | British Columbia, Canada | ancient | Moreno-Mayar et al. 2018(4) , *Science* |
| CK-13 | Ontario, Canada | ancient | Scheib et al. 2018 |
| Sumidouro5 | Brazil | ancient | Moreno-Mayar et al. 2018, *Science* |
| A460 | Chile | ancient | Moreno-Mayar et al. 2018, *Science* |
| I11557 | Santa Rosa Isl, USA | ancient | Nakatsuka et al. 2023(5) |
| I11558 | Santa Rosa Isl, USA | ancient | Nakatsuka et al. 2023 |
| SN-44 | San Nicolas Isl, USA | ancient | Scheib et al. 2018 |
| Pa10A | Panama City, Panama | ancient | Capodiffero et al. 2021(6) |
| Pa16A | Panama City, Panama | ancient | Capodiffero et al. 2021 |
| I1754 | Santiago, Chile | ancient | Posth et al. 2018(7) |
| I2537 | Pica Ocho, Chile | ancient | Posth et al. 2018 |
| CO001 | Culcunche (Cuzco region, Peru) | ancient | Popović et al. 2021(8) |
| CO193 | Cotahuasi (Coropuna volcano, Peru) | ancient | Popović et al. 2021 |
| TW004 | Lake Titicaca, Bolivia | ancient | Popović et al. 2021 |
| TW008 | Lake Titicaca, Bolivia | ancient | Popović et al. 2021 |
| TW020 | Lake Titicaca, Bolivia | ancient | Popović et al. 2021 |
| TW028 | Lake Titicaca, Bolivia | ancient | Popović et al. 2021 |
| TW060 | Lake Titicaca, Bolivia | ancient | Popović et al. 2021 |
| TW097 | Lake Titicaca, Bolivia | ancient | Popović et al. 2021 |
| IL2 | Lake Titicaca, Peru | ancient | Lindo et al. 2018(9) |
| IL3 | Lake Titicaca, Peru | ancient | Lindo et al. 2018 |
| IL4 | Lake Titicaca, Peru | ancient | Lindo et al. 2018 |
| IL5 | Lake Titicaca, Peru | ancient | Lindo et al. 2018 |
| IL7 | Lake Titicaca, Peru | ancient | Lindo et al. 2018 |
| SMP5 | Lake Titicaca, Peru | ancient | Lindo et al. 2018 |
| K1 | Lake Titicaca, Peru | ancient | Lindo et al. 2018 |
| TOY-1-1 | Torontoy (Cuzco region, Peru) | ancient | Nakatsuka et al. 2020(10) |
| TOY-7A-1 | Torontoy (Cuzco region, Peru) | ancient | Nakatsuka et al. 2020 |
| S_Tlingit-1 | Siberia | contemporary | Mallick et al. 2016(11) |
| S_Eskimo_Naukan-1 (Yupik) | Siberia | contemporary | Mallick et al. 2016 |
| S_Mayan-1 | Mexico | contemporary | Mallick et al. 2016 |
| S_Zapotec-1 | Mexico | contemporary | Mallick et al. 2016 |
| S_Karitiana-1 | Brazil | contemporary | Mallick et al. 2016 |
| S_Surui-1 | Brazil | contemporary | Mallick et al. 2016 |
| Loja_Onacapac_01 | Ecuador | contemporary | Joseph et al. 2023(12) |
| Loja_Ganil_04 | Ecuador | contemporary | Joseph et al. 2023 |
| E2_Karanki | Ecuador | contemporary | Joseph et al. 2023 |
| E6_Kisapincha | Ecuador | contemporary | Joseph et al. 2023 |
| S_Quechua-1 | Peru | contemporary | Mallick et al. 2016 |
| 94233 (Aymara) | Bolivia | contemporary | Lindo et al. 2018 |
| Athabaskan 1, 2 | Canada | contemporary | Raghavan et al. 2014(13) |
| Columbia_5000BP | Colombia | ancient | Krettek et al. 2025(14) |
| Columbia_5770BP | Colombia | ancient | Krettek et al. 2025(14) |
| Columbia_1975BP | Colombia | ancient | Krettek et al. 2025(14) |
| Columbia_12988BP | Colombia | ancient | Krettek et al. 2025(14) |
| Columbia_482BP | Colombia | ancient | Krettek et al. 2025(14) |
| Peru_Chincha_Coastal_533BP (UC8_8173) | Peru | ancient | Bongers et al. 2020 (15) |
| Peru_Lambayeque_Coastal_925BP (I2241) | Peru | ancient | Nakatsuka et al. 2020 (10) |
| **Table S1. Published samples used in this study.** | | | |

| ***Sample*** | **Material** | **δ13C,‰** | **14C age years, BP** | **±** | **pMC** | **±** | **Cultural Phase** | **Archaeological Site** |
| --- | --- | --- | --- | --- | --- | --- | --- | --- |
| *SLG-5B* | bioapatite | -4.45 | 2340 | 30 | 74.77 | 0.26 | Very Early Guangala | Salango, Ecuador |
| *SLG-16* | bioapatite | -3.35 | 2250 | 30 | 75.55 | 0.26 | Very Early Guangala | Salango, Ecuador |
| *SLG-27B* | collagen | -9.27 | 740 | 25 | 91.25 | 0.27 | Early Late Manteño | Salango, Ecuador |
| SLG-45B | collagen | -8.54 | 1900 | 20 | 78.92 | 0.22 | Early Guangala | Salango, Ecuador |
| **Table S2: Radiocarbon dating report from the University of Georgia Center for Applied Isotope Studies.** | | | | | | | | |

| **Parameter** | **Description** | **Estimate** | **Prior Range** | **95% CI** | **Units** |
| --- | --- | --- | --- | --- | --- |
| **Effective population sizes (Ne)** | | | | | |
| Ne_Ontario | Effective size of the ancient Ontario population | 11,733 | 500–12,000 | 11,351–11,876 | haploid genomes |
| Ne_Sumidouro | Effective size of Brazil (Sumidouro5, 10.4 ka) | 9,886 | 500–12,000 | 7,703–11,331 | haploid genomes |
| Ne_Colombia | Effective size of Colombia (~5 ka) | 13,934 | 500–16,000 | 11,132–15,391 | haploid genomes |
| Ne_Patagonia | Effective size of Patagonia | 13,192 | 500–16,000 | 10,246–15,312 | haploid genomes |
| Ne_RioUncallane | Effective size of Rio Uncallane (Andes, 1.8 ka) | 12,938 | 500–20,000 | 7,561–17,740 | haploid genomes |
| Ne_SLG | Effective size of Salango (Ecuadorian coast, 2.3 ka) | 719 | 500–12,000 | 645–784 | haploid genomes |
| Ne_Anzick | Effective size of Anzick-1 (Montana, 12.6 ka) | 11,754 | 500–12,000 | 11,447–12,000¹ | haploid genomes |
| Ne_Ontario | | | | | |
| T_RIO_into_SLG | Divergence between Rio Uncallane and Salango | 372 | 360–379 | 367–375 | generations |
| T_SLG_from_SNA | Divergence of Salango from the broader SNA lineage | 392 | 380–440 | 385–404 | generations |
| T_Pat_from_SNA | Divergence of Patagonia from the SNA lineage | 453 | 441–460 | 448–453 | generations |
| T_Brazil_from_SNA | Divergence of Brazil/Sumidouro from the SNA lineage | 473 | 461–480 | 469–475 | generations |
| T_Col_from_SNA | Divergence of Colombia from the SNA lineage | 492 | 481–500 | 487–492 | generations |
| **Older splits (northern lineages)** | | | | | |
| Anzick → Ontario | North American divergence | 640 | fixed |  | generations |
| Ontario → USR1 | Arctic–Beringian divergence | 785 | fixed |  | generations |
| USR1 → MA1 | Arctic–Siberian divergence | 892 | fixed |  | generations |
| MA1 → root | Absolute root event | 1,785 | fixed |  | generations |
| **Table S3. Parameter estimates from the fastsimcoal2 demographic model.** Model includes nine populations (Russia_MA1_24000 BP, Alaska_USR1_11400 BP, Canada_Ontario_2895 BP, Brazil_Sumidouro_10400 BP, Colombia_5000 BP, Patagonia, Rio Uncallane, Salango [SLG], and Anzick-1). Effective population sizes (Ne) are given for haploid genomes; split times are in generations before present, assuming 25 years per generation.  ¹ Six of the 100 bootstrap replicates reached the explicitly enforced upper bound for Ne_Anzick. | | | | | |

**Supplementary Note 1: Fastsimcoal tpl file used for demographic simulation.**

//Number of population samples (demes)

9

//Population effective sizes, haploid (number of genes): Russia_MA1_24000BP, Alaska_USR1_11400BP, Canada_Ontario_2895BP, Brazil_Sumidouro5_10400BP, Colombia_5000BP, Patagonia, Rio_Uncallane, SLG, Anzick-1

3370

2750

Ne_Ontario

Ne_Sumidouro

Ne_Colombia

Ne_Patagonia

Ne_RioUncallane

Ne_SLG

Ne_Anzick

//Samples sizes

2 960 0

2 456 0

2 115 0

2 400 0

2 204 0

2 200 0

2 72 0

2 92 0

2 500 0

//Growth rates

0

0

0

0

0

0

0

0

0

//Number of migration matrices: 0 implies no migration between demes

0

//historical event: time, source, sink, migrants, new size, new growth rate, migr. matrix

9 historical event

T_RIO_into_SLG	6 7 1 1 0 0	// youngest SA split

T_SLG_from_SNA	7 5 1 1 0 0

T_Pat_from_SNA	5 3 1 1 0 0

T_Brazil_from_SNA	3 4 1 1 0 0

T_Col_from_SNA	4 8 1 1 0 0	// oldest SA split (but still < 500)

640	8 2 1 1 0 0	// Anzick → Ontario

785	2 1 1 1 0 0	// Ontario → USR1

892	1 0 1 1 0 0	// USR1 → MA1

1785	0 0 0 3000 0 0 absoluteResize

//Number of independent loci [chromosome]

1 0

//Number of contiguous linkage blocks

1

//per Block: data type, num loci, rec. rate and mut rate + optional parameters

FREQ 1 2e-8 1.25e-8

**Supplementary Note 2: Fastsimcoal est file used for demographic simulation.**

// Parameter estimation file for fastsimcoal2

// Priors and constraints for Ne and split times

[PARAMETERS]

// Effective sizes (haploid)

1 Ne_Ontario	UNIF	500	12000

1 Ne_Sumidouro	UNIF	500	12000

1 Ne_Colombia	UNIF	500	16000

1 Ne_Patagonia	UNIF	500	16000

1 Ne_RioUncallane UNIF	500	20000

1 Ne_SLG	UNIF	500	12000

1 Ne_Anzick	UNIF	500	12000

// Split times (generations)

// Free parameters for South American splits

1 T_RIO_into_SLG	UNIF	360	379 output

1 T_SLG_from_SNA	UNIF	380	440 output

1 T_Pat_from_SNA	UNIF	441	460 output

1 T_Brazil_from_SNA	UNIF	461	480 output

1 T_Col_from_SNA	UNIF	481	500 output

**Supplementary Note 3. Community-engaged research agreement with the Comunidad Ancestral Salango.** A formal authorization (Oficio N° CAS-00323, 2 October 2024) was issued by the Comunidad Ancestral Salango and the Centro de Investigaciones Museo Salango approving the scientific study of ancestral human remains from the site of Salango. The community assembly unanimously required that (i) all final study reports be returned to the Museum and community for custodianship, (ii) all ancestral materials be repatriated to Salango for permanent care, (iii) a protocol be established governing any future transfer of archaeological or human materials to national or international laboratories, and (iv) descendant community consultation be mandatory for all such research. This agreement reflects an ongoing, collaborative partnership grounded in transparency, reciprocity, and respect for ancestral heritage.

**Supplemental References**

1. A. A. Behr, K. Z. Liu, G. Liu-Fang, P. Nakka, S. Ramachandran, pong: fast analysis and visualization of latent clusters in population genetic data. *Bioinform. (Oxf., Engl.)* **32**, 2817–23 (2016).

2. M. Rasmussen, *et al.*, The genome of a Late Pleistocene human from a Clovis burial site in western Montana. *Nature* **506**, 225–229 (2014).

3. J. V. Moreno-Mayar, *et al.*, Terminal Pleistocene Alaskan genome reveals first founding population of Native Americans. *Nature* **488**, 370–9 (2018).

4. J. V. Moreno-Mayar, *et al.*, Early human dispersals within the Americas. *Science* **525**, eaav2621 (2018).

5. N. Nakatsuka, *et al.*, Genetic Continuity and Change Among the Indigenous Peoples of California. *Nature* **624**, 122–129 (2023).

6. M. R. Capodiferro, *et al.*, Archaeogenomic distinctiveness of the Isthmo-Colombian area. *Cell* **184**, 1706-1723.e24 (2020).

7. C. Posth, *et al.*, Reconstructing the Deep Population History of Central and South America. *Cell* **175**, 1185-1197.e22 (2018).

8. D. Popović, *et al.*, Ancient genomes reveal long-range influence of the pre-Columbian culture and site of Tiwanaku. *Sci. Adv.* **7**, eabg7261 (2021).

9. J. Lindo, *et al.*, The genetic prehistory of the Andean highlands 7000 years BP though European contact. *Science Advances* **4**, eaau4921 (2018).

10. N. Nakatsuka, *et al.*, A Paleogenomic Reconstruction of the Deep Population History of the Andes. *Cell* **181**, 1131-1145.e21 (2020).

11. S. Mallick, *et al.*, The Simons Genome Diversity Project: 300 genomes from 142 diverse populations. *Nature* **538**, 201–206 (2016).

12. S. K. Joseph, *et al.*, Genomic evidence for adaptation to tuberculosis in the Andes before European contact. *iScience* **26**, 106034 (2023).

13. M. Raghavan, *et al.*, The genetic prehistory of the New World Arctic. *Science* **345**, 1255832 (2014).

14. K.-L. Krettek, *et al.*, A 6000-year-long genomic transect from the Bogotá Altiplano reveals multiple genetic shifts in the demographic history of Colombia. *Sci. Adv.* **11**, eads6284 (2025).

15. J. L. Bongers, *et al.*, Integration of ancient DNA with transdisciplinary dataset finds strong support for Inca resettlement in the south Peruvian coast. *Proc. Natl. Acad. Sci. United States Am.* **117**, 18359–18368 (2020).
